# MSCs Therapy Reverse the Gut Microbiota in Hypoxia-Induced Pulmonary Hypertension Mice

**DOI:** 10.3389/fphys.2021.712139

**Published:** 2021-08-31

**Authors:** Lingjie Luo, Qinhua Chen, Lei Yang, Zhenxia Zhang, Jihong Xu, Deming Gou

**Affiliations:** ^1^Shenzhen Key Laboratory of Microbial Genetic Engineering, Guangdong Provincial Key Laboratory of Regional Immunity and Disease, Vascular Disease Research Center, Carson International Cancer Center, College of Life Sciences and Oceanography, Shenzhen University, Shenzhen, China; ^2^Key Laboratory of Optoelectronic Devices and Systems of Ministry of Education and Guangdong Province, College of Optoelectronic Engineering, Shenzhen University, Shenzhen, China; ^3^School of Life Sciences and Food Engineering, Hanshan Normal University, Chaozhou, China; ^4^Department of Anesthesiology, Shenzhen University General Hospital, Shenzhen University, Shenzhen, China

**Keywords:** pulmonary hypertension, MSC, gut microbiota, biomarker, vascular

## Abstract

Mesenchymal stem cell (MSC) therapy is a promising therapeutic approach based on its strong effect on pulmonary hypertension (PH) in rats. However, the detailed mechanism of MSC therapy remains unknown. Alterations in the gut microbiota were found in both type 1 pulmonary arterial hypertension patients and hypoxia/SU5416- or monocrotaline (MCT)-induced PH rats. However, whether the therapeutic mechanism of MSCs is associated with the gut microbiota is poorly understood. Here, we found that gut microbiota homeostasis was disrupted in hypoxia-induced PH mice due to the increased *Firmicutes*-to*-Bacteroidetes (F/B)* ratio; enhanced abundances of harmful *Marinifilaceae*, *Helicobacteraceae*, and *Lactobacillaceae*; and decreased abundances of beneficial *Bacteroidaceae*, *Prevotellaceae*, *Tannerellaceae*, and *Lachnospiraceae*. Unexpectedly, reverses of the increase in disease-associated microbiota and decrease in anti-inflammatory and immunomodulatory functional microbiota were observed in the MSC-treated group. We also identified harmful *Erysipelotrichaceae*, *Alphaproteobacteria*, *Christensenella timonensis*, *Coriobacteriales*, and *Rhodospirillales* that may serve as gut microbiota biomarkers of hypoxia-induced PH mice. *Micrococcaales*, *Nesterenkonia*, *Anaerotruncus*, and *Tyzzerella* may serve as gut microbiota biomarkers of MSC-treated mice. In summary, MSC treatment suppresses hypoxia-induced pulmonary hypertension in mice, and alterated gut microbiota may play a role in the development and progression of PH. The mechanism of MSC therapy is associated with various metabolic pathways of the gut microbiota in hypoxia model PH mice.

## Introduction

Pulmonary hypertension (PH) is a severe lethal disease with few available drugs. Vasoconstriction, remodeling of the pulmonary vessel wall, inflammation, and thrombosis *in situ* result in the progressive increase of pulmonary vascular resistance and elevated pulmonary arterial pressure which will ultimately lead to right heart failure and death (Pulido et al., 2013). During the past few decades, several drugs have been used to treat pulmonary arterial hypertension (PAH) by targeting the nitric oxide (NO) signaling pathway, prostacyclin pathway and endothelin pathway. These drugs are implicated in vascular constriction, for example, prostacyclin analogs and derivatives (Del Pozo et al., 2017), endothelin receptor antagonists (Liu et al., 2009), phosphodiesterase type 5 (PDE5) inhibitors (Barnes et al., 2019), and soluble guanylate cyclase (sGC) stimulators (Beghetti et al., 2019). However, these drugs have shown limited therapeutic effects on PH. Therefore, it is important to develop new methods targeting other pathological processes associated with the pathophysiology of PAH.

Pulmonary arterial hypertension (PAH) is a multifactorial disease and is implicated in multiple molecular mechanisms. Recent studies have shown that innate immunity and adaptive immunity are involved in PAH by driving the development of PAH. Significant infiltration of macrophages, monocytes, mast cells, dendritic cells, T cells, cytotoxic T cells, and helper T cells was clearly observed in vessels of a rodent PH model and in idiopathic PAH lungs; nevertheless, FoxP31 mononuclear cells were significantly decreased (Savai et al., 2012). The cytokines and inflammation mediators IL-6, TNF-α, IL-1β, IL-8, and MCP-1 were increased in PAH patient lung tissues and peripheral blood (Satoh et al., 2014). Elevated levels of chemokines, such as CCL2, CCL4, CX3CL1, and CXCL10, were also observed in PAH (Rabinovitch et al., 2014). The innate immunity NF-κB signaling pathway is activated in PAH patients and rodent PH models. The complement cascade was also identified as a consistent critical determinant of PH in rodent models and humans (Frid et al., 2020). IL-6 overexpression can promote the development of PH (Steiner et al., 2009). Recombination–activating gene (RAG) proteins initiated V(D)J recombination of immunoglobulin and T cell receptor genes during B and T lymphocyte development in jawed vertebrates (Huang et al., 2016). *RAG1*^–/–^ C57 mice had diminished right ventricular systolic pressure and arterial remodeling compared with those in wild-type mice exposed to chronic hypoxia (Maston et al., 2017). These results indicate that innate immunity and adaptive immunity play important roles in PH.

The intestinal microbiota plays a crucial role in influencing the development of host immunity, and the immune system also regulates the microbiota through the intestine. The association of intestinal microbiota and innate/adaptive immunity is in dynamic equilibrium and tightly regulated (Brown et al., 2019). The gastrointestinal tract tissues have the unique property of harboring an enormous number of microbes within the lumen. These tissues harbor hundreds of species of microbiota that intimately interact with the hosts and provide them with genetic, metabolic, and immunological attributes (Lozupone et al., 2012). Recent reports have indicated that the composition of the microbiota and its collective genome (the microbiome) are major factors in predetermining the type and robustness of mucosal immune responses (Caballero and Pamer, 2015). Alterations in the human intestinal microbiota are associated with inflammatory diseases, such as inflammatory bowel disease (IBD) (Nishida et al., 2018), irritable bowel syndrome (IBS) (Bhattarai et al., 2017), colorectal cancer (Wong and Yu, 2019), autoimmune and metabolic diseases, such as type I diabetes, type 2 diabetes (Bolla et al., 2019), rheumatoid arthritis (Horta-Baas et al., 2017), obesity, and cardiovascular disease (Wang and Zhao, 2018). The intestinal microbial community is changed and loses its balance in iPAH patients (Kim et al., 2020). Monocrotaline (MCT)-induced rat PH showed a profound gut pathology, which was associated with alterations in microbial communities, some of which were unique to PH animals (Sharma et al., 2020). Alternative gut microbes were also observed in hypoxia/Sugen 5416-induced PH rats (Callejo et al., 2018). Koichiro Tatsumi’s group indicated that the development of PH was suppressed by the gut microbiota of antibiotic-induced modification (Sanada et al., 2020). This evidence indicates that an imbalance of the gut microbiota may play an important role in the pathogenesis of PH.

Human umbilical cord blood-derived mesenchymal stem cells (hUC-MSCs, hereafter referred to as MSCs) are multiple functional cells that can sense harsh environments and secrete proteins to respond to abnormal environments (Bagno et al., 2018). Over the past few decades, MSCs have been used to treat numerous diseases, such as pulmonary fibrosis and chronic obstructive pulmonary disease, because of their immunoregulatory function (Trounson and McDonald, 2015; Naji et al., 2019; Golchin et al., 2020). MSCs were reported to be able to treat MCT-induced rat PH and had no side effects, and the pre-inflammatory cytokines IL-6, IL-1β, and TNF-α were decreased in MSC-treated rats after MCT injection (Kim et al., 2016). Researchers have indicated that MSC therapy are associated with the gut microbiota in many diseases, for example, IBD (Soontararak et al., 2018), acute liver injury (Dong et al., 2019), and chronic hypoxia, which leads to the gut dysbiosis (Xing et al., 2018). However, whether MSCs have therapeutic functions in hypoxia-induced mice is not clear, and whether the molecular mechanism of MSC therapy is associated with gut microbiota remains unknown. Given that inflammation plays an important role in PH, MSCs therapy decreases inflammation in MCT-induced PH rats, the gut microbiota is disordered in PH, and the immune system interacts with the gut microbiota, we sought to explore whether the gut microbiota was associated with MSC treatment for PH. For example, we sought to determine whether a specific gut microbe could serve as a biomarker of PH mice and MSC-treated mice and whether the gut microbiota is changed or restored to normal in MSC-treated PH mice.

Here, we showed that MSC therapy could attenuate hypoxia-induced PH in mice. We also analyzed the intestinal microbial community in hypoxia-induced PH mice and MSC-treated mice through 16S rRNA sequencing. The results indicated that the gut microbiota was disordered in hypoxia-induced mice and that the changes in hypoxia-induced mice were reversed in MSC-treated mice.

## Materials and Methods

### Animals and Reagents

Eight-week-old male wild-type ICR mice (weight, 20–25 g) (Guangdong Medical Laboratory Animal Center, Guangzhou, China) were used to establish hypoxia-induced PH and MSC therapy. Wild-type ICR mice were housed in a barrier environment at 22°C. All animals received humane care, and all procedures were approved by the Animal Care and Use Committee of the Shenzhen University.

### MSC-Based Therapy for PH

Twenty-three mice were divided into three groups: the control mice received a PBS treatment (*n* = 8); The hypoxic mice were exposed to hypoxia in 10% O_2_ for 3 weeks (*n* = 8); the MSC groups’ mice were tail-intravenous injection with 1 × 10^6^ MSCs per mouse before hypoxia (*n* = 7). The potential curative effect of MSCs was evaluated according to biochemical and pathological results.

### Cell Culture

MSCs were obtained from Shenyang Lianxing Biotechnology Co., Ltd., Shenyang, Liaoning, and cultured in DMEM/F12 with 10% (vol/vol) fetal bovine serum (Gibco) at 37°C and 5% CO_2_ in an incubator (Thermo Fisher Scientific Inc., Waltham, MA, United States). The phenotype of the MSCs was investigated by fluorescence-activated cell sorting (FACS) (Supplementary Figure 1). FACS results showed that MSCs were positive for CD90 (99.90%), CD105 (100%), and CD73 (99.8%) and negative for CD45 (0.3%), CD34 (0%). This study was approved by the Institutional Review Board of the Shenzhen University General Hospital and the Institutional Review Board of the Shenzhen University School of Medicine according to the established ethical guidelines as outlined in the Declaration of Helsinki.

### 16S rRNA Gene Amplification and Multiparallel Sequencing

Fecal samples originating from normoxia- and hypoxia-induced MSC-treated mice were immediately frozen at −80°C. Total genomic DNA from the samples was extracted using a QIAGEN DNA extraction kit. The 16S rRNA genes of the 16S V3-V4 regions were amplified using specific primers with barcodes. PCR was carried out with Phusion High-Fidelity Taq Enzyme (*NEB*) following the manufacturer’s recommendations. Then, the 16S V3-V4 regions were purified with a DNA Gel Extraction Kit (Promega) following the manufacturer’s recommendations. An Ion Plus Fragment Library Kit (48 rxns; Thermo Fisher Scientific) was used to generate sequencing libraries following the manufacturer’s recommendations. Library quality was assessed on a Qubit@ 2.0 Fluorometer (Thermo Fisher Scientific). Finally, the libraries were sequenced on the Ion S5^TM^ XL platform, and 400 bp/600 bp single-end reads were generated. The raw data were deposited in the NCBI public database (accession number: SRR14292713-SRR14292733).

### Immunofluorescence and Confocal Microscopy

Lung tissues were fixed in formalin and embedded in paraffin (Leica), and 6 nm sections of lung tissues were deparaffinized and permeabilized with xylol and then incubated with a mouse anti-α-SMA antibody, goat anti-mouse IgG, and DAPI. Briefly, the sections were blocked with PBS containing 5% Tween and 5% bovine serum albumin (Sangon Biotech Corp.) for 45 min at room temperature. Then, the sections were incubated with a mouse anti-α-SMA antibody (1:1,000; Sigma) overnight at 4°C and washed three times with PBST. Subsequently, the sections were incubated with FITC-conjugated goat anti-mouse IgG (1:500; Invitrogen Life Technologies) for 60 min and washed three times with PBST. Nuclei were stained with 4′,6-diamidino-2-phenylindole (DAPI) for 10 min and washed three times with PBS. A confocal microscope system was used to examine pulmonary artery (PA) remodeling (Zeiss LSM-710, Germany). Images were acquired using ZEN2.3 software.

### Histological Analysis

Lung tissues were fixed in formalin and embedded in paraffin (Leica), and 6 nm sections of lung tissues were deparaffinized and permeabilized with xylol and then stained with hematoxylin and eosin dyes. The sections were then examined under a microscope. The remodeling of the pulmonary vasculature was quantified by calculating the medial wall thickness and the ratio of the medial wall area (MWA) to the total vessel cross sectional area (CSA) by ImageJ software (National Institutes of Health, Bethesda, MD, United States).

### Quantitative Real-Time PCR

Total RNAs of lung tissues were extracted using TRIzol reagent (TAKARA) and first-strand cDNA was synthesized with Oligo(dT) primers (TAKARA) according to the manufacturer’s instructions. qRT-PCR was performed in Light Cycler 1.5 (Roche Applied Science, Penzberg, Germany). Primers were described in Supplementary Table 2.

### The Statistical Analysis

The results are expressed as the means ± SEM from at least five independent experiments. Significant differences between two or multiple different experimental groups were determined using Student’s *t*-test.

## Results

### MSCs Prevent Hypoxia-Induced PH by Attenuating Pulmonary Artery Remodeling

**FIGURE 1:**
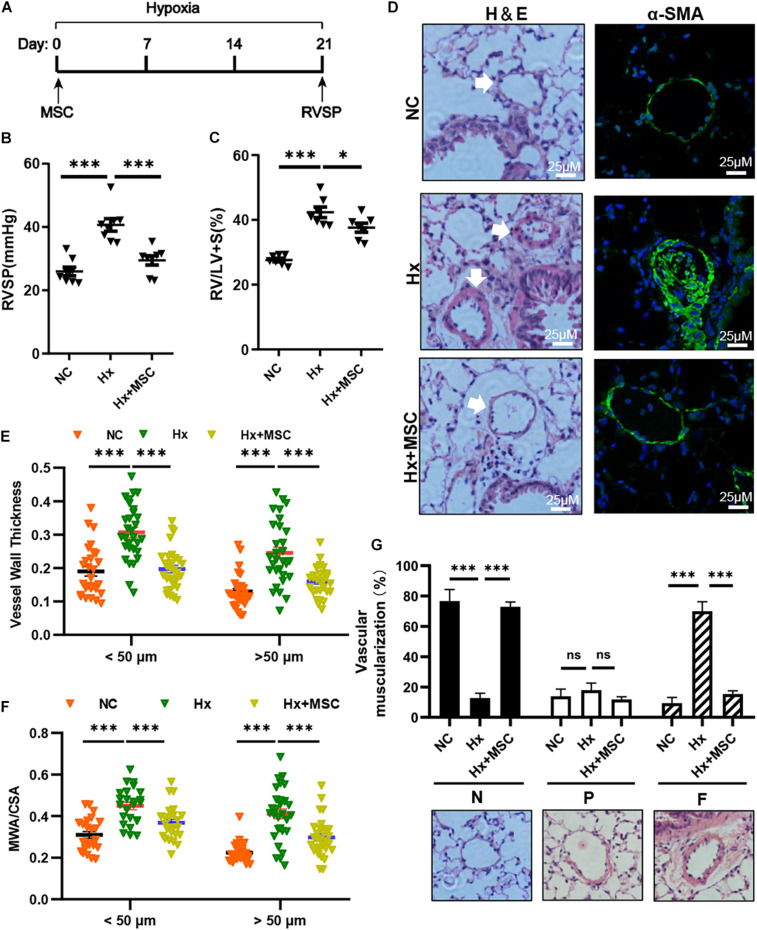
MSCs attenuate the development of hypoxia (Hx)-induced PH in mice. **(A)** Protocol for the administration of MSCs to Hx-challenged mice. **(B,C)** Effect of MSC administration on RVSP and the RV/LV + S ratio in hypoxia-induced PH mice. ^∗^*P* < 0.05, ^∗∗∗^*P* < 0.001 (*n* = 7). **(D)** Representative hematoxylin and eosin-stained lung sections and immunofluorescence images of α-SMA (green) expression in Hx-induced mice treated with MSCs. Scale bars: 25 μm. **(E)** Quantification of the vessel wall thickness of the pulmonary artery. ^∗∗∗^*P* < 0.001. **(F)** Quantification of the ratio of the medial wall area (MWA) to the total vessel cross sectional area (CSA). ^∗∗∗^*P* < 0.001. **(G)** Proportion of non- (N), partially (P), or fully (F) muscularized PAs in the total counted PAs. A total of 30–50 vessels were analyzed in each mouse (*n* = 4–6), ^∗∗∗^*P* < 0.001. Data are presented as the mean ± SEM. Statistical significance was evaluated by *t*-tests.

To evaluate the therapeutic effect of MSCs on hypoxia-induced PH (Hx, 10% O_2_, 21 days), we transplanted mice with 1 × 10^6^ human MSCs *via* tail vein injection (Figure 1A). Compared with those in the Hx groups injected with the PBS control, the MSC-treated groups showed significant decreases in RVSP and RV/(LV + S) (Figures 1B,C). Histological and immunofluorescence staining showed significant increases in the pulmonary vascular wall area and thickness after 21 days of chronic hypoxia exposure, which were largely attenuated by MSC treatment (Figures 1D–F). The vascular muscularization degree was increased in hypoxia-induced mice, and the muscularization of PAs was blocked in MSC-treated mice (Figure 1G). These results showed that MSCs could effectively attenuate hypoxia-induced mouse PH.

### Overall Microbial Content Under Conditions of Normoxia and Hypoxia and MSC Treatment

**FIGURE 2:**
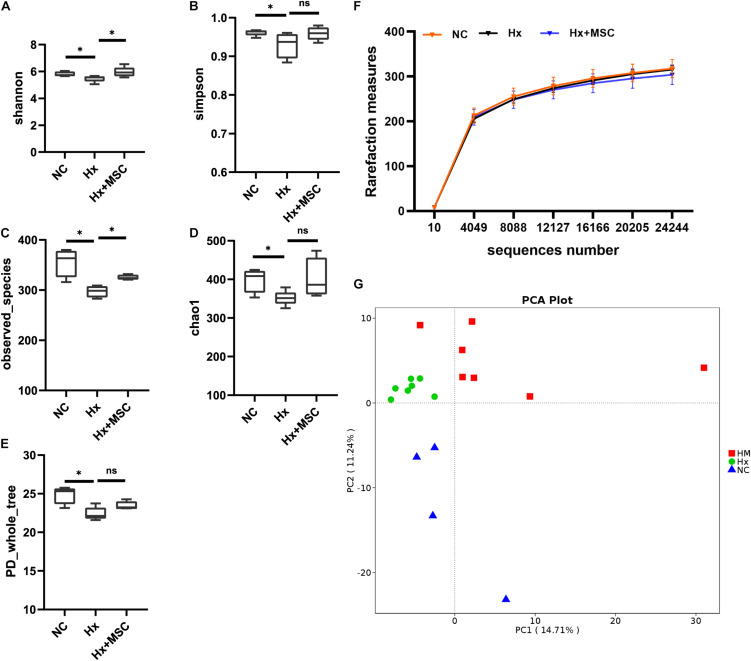
MSC treatment reverses the gut microbiota structure in hypoxia-induced PH mice. Feces was collected from Kunming mice at 3 weeks. **(A–E)** The α-diversity of gut microbiota in normoxia-, hypoxia-induced and MSC-treated mice measured by the Shannon **(A)**, Simpsons **(B)**, Chao1 **(C)**, PD whole tree **(D)**, observed_species **(E)** indices. ^∗^*P* < 0.05. The results are presented as means ± SEM of 4–7 animals. Chao1 estimates the number of species, whereas Shannon estimates the effective number of species. **(F)** Rarefaction curves for the observed species in the gut microbiota from normoxia-, hypoxia-induced, and MSC-treated mice. *N* = 4–7; **(G)** principal component analysis. NC, normoxia group; Hx, hypoxia-induced group; Hx + MSCs, MSC-treated group.

To reveal the possible links between the effects of MSC therapy and the gut microbiota, prokaryotic 16S ribosomal RNA (rRNA) in the variable V3-V4 region of fecal samples from the normoxia groups (NC, *n* = 7), hypoxia-induced group (Hx, *n* = 7), and MSC-treated group (Hx + MSCs, *n* = 7) was sequenced. Based on the unique sequences of each sample and the specific filtering conditions, a total of 1610484 [560815 for the normoxia group (NC), 560700 for the hypoxia-induced group (Hx), 510518 for the MSC-treated group (Hx + MSC)] high-quality clean reads (accession number: SRR14292713-SRR14292733) were acquired from all the samples, with an average of 76,689 (range: 54,753–80,236) reads per sample used for downstream statistical analysis. All the sequences were clustered into 526 operational taxonomic units (OTUs). Specifically, there were 472 OTUs in the NC group, 485 OTUs in the Hx group and 467 OTUs in the Hx + MSC group. More than 99.5% of all the samples had good coverage, indicating sufficient community coverage. The details are shown in Supplementary Table 1. The Hx group had decreased alpha diversity (α-diversity) values (measured by the Shannon and Simpson indices) and richness indices (measured by Chao1 and the observed species). The Hx + MSC group demonstrated a significant reversal of the reduced α-diversity values and richness indices in the Hx group (Figures 2A–E). The rarefaction curves of the observed species approached a plateau, indicating that the sequencing was sufficient in all samples and covered all the OTUs (Figure 2F). The rank abundance curves fell slowly, indicating that the samples were not dominated by a few OTUs but mostly by low-abundance OTUs (Supplementary Figure 2A). Alterations in the microbiota composition of all the groups and samples were noted according to PcoA (Supplementary Figure 2B). Additionally, principal component analysis (PCA) showed that the samples from normoxia-, hypoxia-induced, and MSC-transplanted groups were separated and clustered together (Figure 2G).

### Alterations in the Gut Microbiota in Response to Hypoxia-Induced PH and MSC Treatment

**FIGURE 3:**
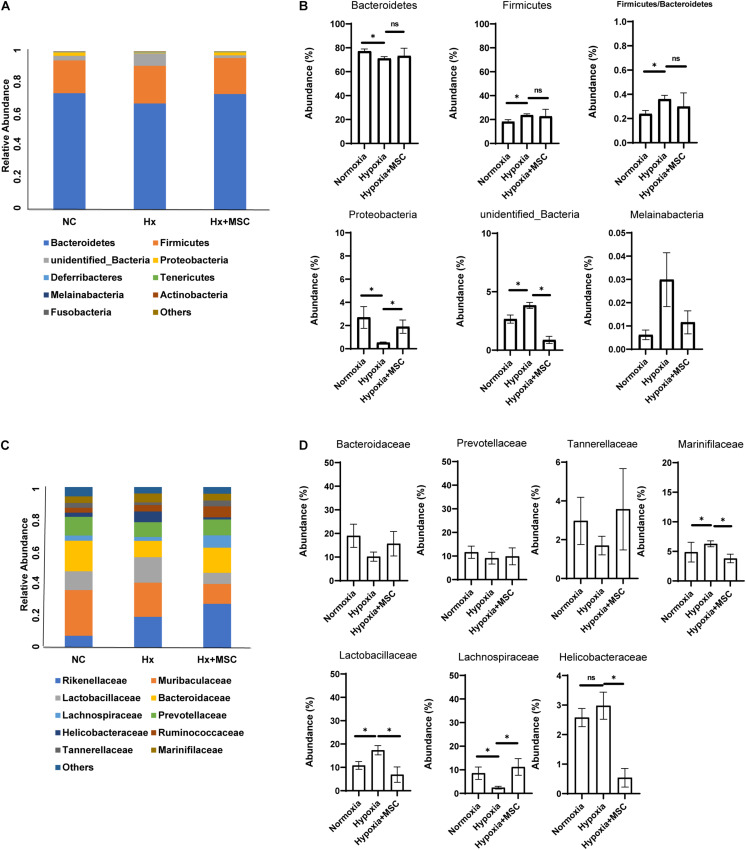
Fecal microbial composition at the phylum level and family level. **(A,B)** The changes in *Firmicutes*, *Bacteroidetes*, *Proteobacteria*, unidentified_bacteria, *Melainabateria*, and the ratio of *Firmicutes/Bacteroidetes*. *n* = 4–5 per group; mean ± SEM; White’s non-parametric *t*-test, ^∗^*P* < 0.05. **(C,D)** The most abundant taxon changes at the family level. *n* = 4–5 per group; mean ± SEM; White’s non-parametric *t*-test, ^∗^*P* < 0.05.

To determine the changes in the gut microbiota that are associated with hypoxia-induced PH and administration of MSCs, we analyzed the differences in the gut microbiota among the three groups. At the phylum level, compared to those in the normoxia group, the abundances of *Firmicutes*, *unidentified_Bacteria*, and *Melainabacteria* were significant increased; the abundances of *Bacteroidetes* and *Proteobacteria* were significantly decreased; and the ratio of *Firmicutes*/*Bacteroidetes* was increased in hypoxia-induced group. Compared with those in the hypoxia-induced group, the MSC-treated groups exhibited increases in the abundances of *Bacteroidetes* and *Proteobacteria* but decreases in the abundances of *Firmicutes*, *unidentified bacteria*, and *Melainabacteria* (Figures 3A,B). Furthermore, at the family level, the families *Lactobacillaceae*, *Bacteroidaceae*, *Prevotellaceae*, *Marinifilaceae*, and *Tannerellaceae* were the most abundant representatives of the *Bacteroidetes* phylum (Figure 3B). *Lachnospiraceae* were the most abundant representatives of the *Firmicutes* phylum. The family *Helicobacteraceae* was the most abundant representative of the *Proteobacteria* phylum. Overall, compared with those in the normoxia group, the abundances of *Bacteroidaceae*, *Prevotellaceae*, and *Tannerellaceae* were decreased in the hypoxia-induced group, the MSC-treated group reversed the reduction of these families and there were no statistically significant differences. *Lachnospiraceae* were significantly decreased in the hypoxia-induced group, and the MSC-treated group significantly reversed this reduction. The MSC-treated group reversed the increases in *Marinifilaceae*, *Helicobacteraceae*, and *Lactobacillaceae* in hypoxia-induced mice (Figures 3C,D). The above results demonstrate that the MSC treatment reversed disorders of the gut microbiota in the hypoxia-induced group.

**FIGURE 4:**
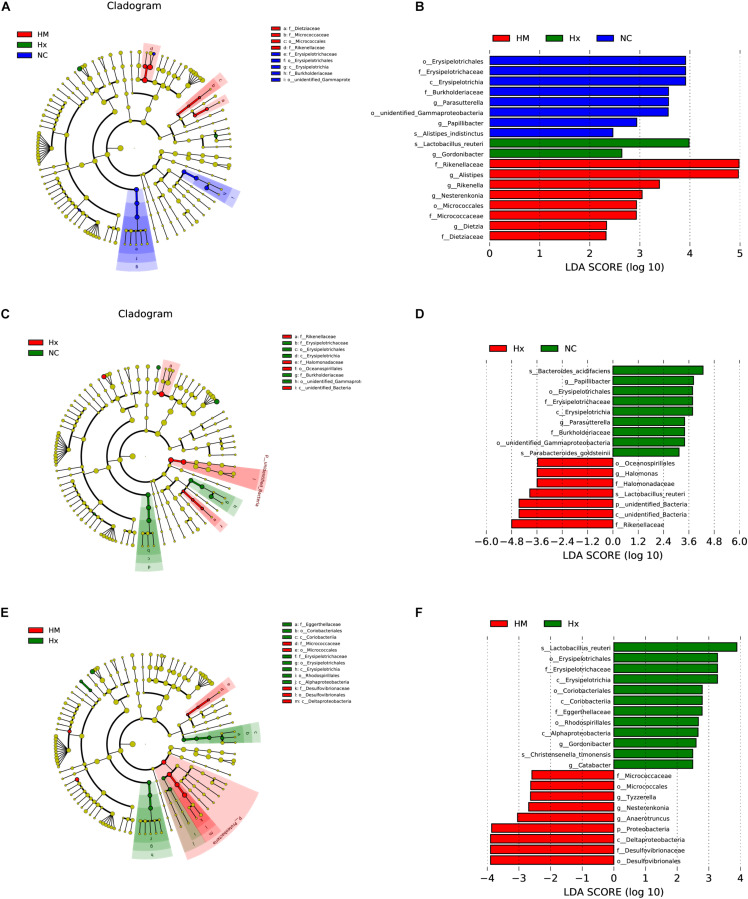
LDA of the microbial community variation. **(A)** The cladogram represents the phylogenetic relationship of significant OTUs associated with NC, Hx and HM. **(B)** Linear discriminant effect size (LEfSe) analysis was performed (alpha value ≥ 0.05, logarithmic LDA score threshold ≥ 2) on all three groups together. **(C)** The cladogram represents the phylogenetic relationship of the significant OTUs associated with NC and Hx. **(D)** Linear discriminant effect size (LEfSe) analysis was performed (alpha value ≥ 0.05, logarithmic LDA score threshold ≥ 2) on NC and Hx. **(E)** The cladogram represents the phylogenetic relationship of the significant OTUs associated with Hx and HM. **(F)** Linear discriminant effect size (LEfSe) analysis was performed (alpha value ≥ 0.05, logarithmic LDA score threshold ≥ 2) on Hx and HM. NC, Vehicle control; Hx, hypoxia-induced group; HM, MSC-treated group. *n* = 4–5.

To identify biomarkers for the hypoxia-induced group and MSC-treated group, the differences in microbial components among all the groups, as well as between selected groups, were compared by linear discriminant analysis and linear discriminant effect size (LEfSe). When the gut microbiota of all three groups was analyzed together, 18 total discriminative features were identified. *Erysipelotrichaceae* of the *Firmicutes* phylum; *Burkholderiaceae*, *unidentified_Gammaproteobacteria*, and *Parasutterella* of the *Proteobacteria* phylum; *Papillibacter* and *Alistipes_indistinctus* of the *Bacteroidetes* phylum; and *Papillibacter* were discriminative in normoxic mice. *Lactobacillus* of the *Firmicutes* phylum and *Gordonibacter* of the *Actinobacteria* phylum were discriminative in hypoxia-induced PH mice. *Alistipes* and *Rikenellaceae* of the *Bacteroidetes* phylum, Nesterenkonia of the *Actinobacteria* phylum, and *Dietziaceae* were discriminative in MSC-treated mice (Figures 4A,B).

In addition to the microbial components identified upon analysis of all the groups together, we also analyzed differences in the microbial components between two specific groups. When we analyzed normoxia and hypoxia alone, *Bacteroides_acidifaciens* and *Parabacteroides_goldsteinii* of the *Bacteroidetes* phylum were identified in normoxic mice, and *Oceanospirillales* and *Halomonas* of *Proteobacteria*, *Rikenellaceae* of the *Bacteroidetes* phylum, and *unidentified_bacteria* were predominant in the hypoxia-induced group (Figures 4C,D). When we analyzed hypoxia and the MSC treatment alone, *Christensenella timonensis* and *Erysipelotrichaceae* of the *Firmicutes* phylum, *Coriobacteriales* of the *Actinobacteria* phylum, *Alphaproteobacteria* and *Rhodospirillales* of the *Proteobacteria* phylum, and *Eggerthellaceae* were enriched in the hypoxia-induced group. *Anaerotruncus* and *Tyzzerella* of the *Firmicutes* phylum and *Desulfovibrionaceae* of the *Proteobacteria* phylum were enriched in the MSC-treated group (Figures 4E,F).

### Potential Functions of the Gut Microbiota in the Hypoxia-Induced and MSC-Treated Groups

**FIGURE 5:**
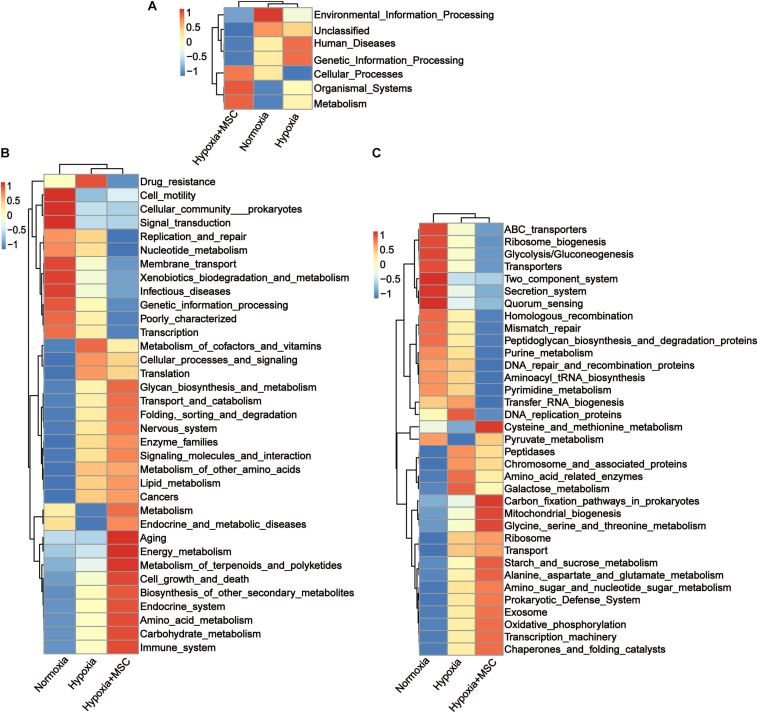
Kyoto Encyclopedia of Genes and Genomes (KEGG) pathway analysis at level 1 **(A)**, level 2 **(B)**, and level 3 **(C)**. Statistically significant KEGG pathways of the normoxia, hypoxia-induced and MSC-treated groups were determined by STAMP software, and LEfSe (LDA score > 2) of significant KEGG pathways was performed.

To reveal the potential function of the gut microbiota in MSC-treated PH mice, Kyoto Encyclopedia of Genes and Genomes (KEGG) analysis was performed. Figure 5 shows the predicted pathways at level 1 (A), level 2 (B), and level 3 (C) in the normoxia-, hypoxia-induced, and MSC-treated groups. At level 1, the gut microbiota of the hypoxia-induced group was mainly involved in human diseases and genetic information processing, while the intestinal microbiota of the MSC-treated group was mainly involved in metabolism, organismal systems and cellular processes (Figure 5A). At level 3, transfer RNA biogenesis, DNA replication proteins, peptidases, chromosome and associated proteins, amino acid-related enzymes, galactose metabolism, ribosome, transport, DNA repair and recombination proteins, pyrimidine metabolism, purine metabolism, homologous recombination, mismatch repair, peptidoglycan biosynthesis and degradation proteins, and aminoacyl tRNA biosynthesis pathways were mainly found in the hypoxia-induced group. Cysteine and methionine metabolism; carbon fixation pathways in prokaryotes; mitochondrial biogenesis; glycine, serine and threonine metabolism; starch and sucrose metabolism; alanine, aspartate and glutamate metabolism; amino sugar and nucleotide sugar metabolism; prokaryotic defense system; exosomes; oxidative phosphorylation; transcription machinery; chaperones; and folding catalyst pathways were mainly found in MSC-treated group (Figure 5C).

## Discussion

**FIGURE 6:**
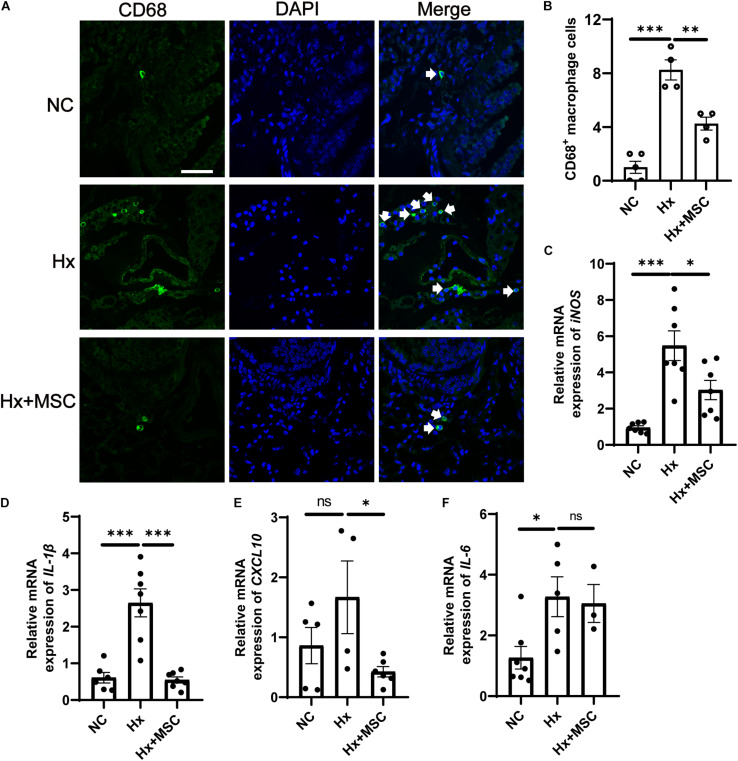
Administration of MSC reduces inflammation in hypoxia-induced PH. **(A)** Immunofluorescence images of DAPI (blue) and CD68 (green) expression in hypoxia-induced mice treated with MSCs. White arrow: CD68^+^ macrophage. **(B)**, Quantification of CD68^+^ macrophages in the lungs of mice. A total of 10 fields of view per mouse were counted. ^∗∗∗^*P* < 0.001. NC, Vehicle control; Hx, hypoxia-induced group; Hx + MSC, MSC-treated group. *n* = 4–5; Scale bars = 50 μm. **(C–F)** The mRNA level of *iNOS*, *IL-1*β, *IL-6*, and *CXCL10* in lung tissues from NC, Hx, MSC groups’ mice 21 days after corresponding treatment were detected by qRT-PCR. ^∗^*P* < 0.05, ^∗∗^*P* < 0.01, ^∗∗∗^*P* < 0.001. NC, Vehicle control; Hx, hypoxia-induced group; Hx + MSC, MSC-treated group. *n* = 3–7.

Mesenchymal stem cells (MSCs) and conditioned medium from MSCs were used to treat MCT-induced rat PH (Lee H. et al., 2015; Lee J. C. et al., 2015). A subset of genes involved in various pathophysiological processes was changed in MSC-treated PH rats after MCT induction according to mRNA-seq (Lee et al., 2014). We deduced that MSCs may attenuate hypoxia-induced PH in mice. Indeed, MSCs significantly attenuated the development of hypoxia-induced PH in mice by attenuating PA remodeling (Figure 1). PA remodeling and inflammation in lung tissues are the main characteristics of PH. PA remodeling and the levels of the pre-inflammatory cytokines IL-6, IL-1β, and TNF-α were reversed in MSC-treated rats after MCT injection (Kim et al., 2016). Our results indicated that CD68^+^ macrophages were increased in hypoxia-induced mice and that MSC therapy reversed the level of CD68^+^ macrophages (Figures 6A,B). The mRNA expression of cytokines *IL-6*, *IL-1*β, and *iNOS* were increased in hypoxia-induced mice, while *IL-1*β and *iNOS* were reversed by MSC administration in hypoxia-induced PH mice. These results indicated that MSC therapy can attenuate mouse PH by decreasing inflammation (Figures 6C–F).

In recent years, research has indicated that MSC therapy is associated with the gut microbiota in many diseases. Compared to that in dextran sulfate sodium (DSS)-treated animals, the gut microbiota in iMSC-treated animals was more similar to that in control animals (Soontararak et al., 2018). Chronic hypoxia leads to the senescence of bone marrow mesenchymal stem cells (BMSCs) and gut dysbiosis (Xing et al., 2018). MSC therapy for IBD has also been implicated in involving the gut microbiota, and gastrointestinal bacteria are capable of inducing immune-regulatory mediator secretion, cytokine gene transcription and surface protein expression in MSCs (Ocansey et al., 2019). The previous studies demonstrate that there is some association between MSC therapy and the gut microbiota.

The gut microbiota contains large numbers of microorganisms with various functions. The gut microbiota interacts with the host to maintain homeostasis, and an imbalance can result in disease. *Firmicutes* and *Bacteroidetes* (*F/B*) are the most abundant bacterial phyla affecting host physiology in both humans and mice. An imbalanced *F/B* ratio is associated with various disease processes (Liu et al., 2016). MCT-induced PH rats showed an increased *F/B* ratio, suggesting that the F/B ratio can serve as an indicator of PH (Sharma et al., 2020). Our results also showed that the ratio of *Firmicutes/Bacteroidetes* was increased in hypoxia-induced PH mice and that MSC treatment reversed this imbalance, which indicated the correlation between MSC treatment and the gut microbiota (Figure 3B). *Helicobacteraceae*, which belongs to the *Proteobacteria* phylum, is reported to be associated with gastrointestinal tract diseases in animals (Whary and Fox, 2004). The MSC-treated group reversed the increase in harmful *Helicobacteraceae*, *Marinifilaceae*, and *Lactobacillaceae* in hypoxia-induced mice (Figures 3C,D). At the same time, we found that *Lachnospiraceae* and *Prevotellaceae* were decreased in hypoxia-induced mice, and MSC-treated mice exhibited the opposite reduction. *Lachnospiraceae* and *Prevotellaceae* are short-chain fatty acid (SCFA)-producing bacteria (Kim et al., 2014). SCFAs inhibit the inflammatory response by inhibiting the NF-κB pathway in macrophages and increase the production of IL-10 by T cells (Furusawa et al., 2013). Macrophages were decreased after MSC treatment (Figure 6), suggesting that MSCs may ameliorate PH *via* the gut microbiota—SCFAs–macrophages—IL-6/TNF-α axis. Furthermore, *Lachnospiraceae* are butyrate-producing bacteria, and butyrate performs multiple functions by inhibiting the activity of histone deacetylases (HDACs) (Silva et al., 2018; Chen et al., 2019; Jaworska et al., 2019). HDAC inhibitors can attenuate MCT-induced rat PH by suppressing the expression of *Nox* (Chen et al., 2016). These results suggested that MSCs may ameliorate PH *via* the *Lachnospiraceae*-butyrate-HDAC-Nox axis. We also found that *Bacteroidaceae* and *Tannerellaceae* were decreased in the hypoxia-induced group, and the MSC-treated group exhibited the opposite reduction. All of these results indicated that the gut microbiota of MSC-treated mice tended to be normal and that MSC-treated mice may be able to overcome PH *via* regulation of the gut microbiota.

Principal component analysis (PCA) showed that the samples from the normoxia-, hypoxia-induced, and MSC-treated groups were clustered together within groups and separated from each other, and each group contained a specific gut microbiota (Figure 2G). These findings indicated that our system was reliable and could be used to screen biomarkers.

Some predominant bacteria can serve as biomarkers of normal or disease; for example, *Parabacteroides_goldsteinii* and *Bacteroides_acidifaciens* have anti-obesity effects (Yang et al., 2017; Wu et al., 2019). In our system, we also identified the beneficial *Parabacteroides_goldsteinii* and *Bacteroides_acidifaciens* as predicted biomarkers of normoxic mice, while harmful *Oceanospirillales*, *Halomonas*, and *Rikenellaceae* were predicted as biomarkers of hypoxia-induced mice, indicating that the prediction system was reliable and could be used to screen biomarkers *via* LEfSe analysis of normoxia and hypoxia alone (Figures 4C,D). Dinh’s group observed that the relative abundance of *Erysipelotrichi* was positively correlated with tumor necrosis factor alpha (TNF) levels in a study investigating patients who had a chronic HIV infection and were receiving suppressive antiretroviral therapy and HIV-uninfected controls (Dinh et al., 2015; Kaakoush, 2015). *Alphaproteobacteria* can trigger autoimmune disease (Mohammed and Mattner, 2009). We also identified harmful *Erysipelotrichaceae*, Alphaproteobacteria, *Christensenella_timonensis* (Ndongo et al., 2016), Coriobacteriales (Picchianti-Diamanti et al., 2018), Rhodospirillales (Rosales et al., 2020), and Eggerthellaceae as predicted biomarkers of the hypoxia-induced group. *Anaerotruncus* and *Tyzzerella* were predicted biomarkers of the MSC-treated group when LEfSe analysis was performed between the hypoxia-induced group and the MSC-treated group (Figures 4E,F). Thus, these biomarkers may help to predict disease predisposition, activity, severity, and responsiveness to therapy.

The gut microbiota has collective metabolic and immunoregulation abilities that are relevant to host health and disease (Caballero and Pamer, 2015). The potential functions of the gut microbiota in the MSC-transplanted group differed significantly from those in hypoxia-induced mice. The gut microbiota of the hypoxia-induced group was mainly involved in human diseases and genetic information processing, and the function of the gut microbiota was in accordance with the harmful microbiota biomarkers identified by LEfSe analysis in hypoxia-induced mice. The intestinal microbiota of the MSC-treated group was mainly involved in metabolism, organismal systems and cellular processes (Figure 5C). This finding was in accordance with the increase in SCFA-producing bacteria in the MSC-treated group.

An emerging metabolic theory of PH suggests that cellular and mitochondrial metabolic dysfunction underlies the pathology of this disease (Zhang et al., 2017). Aerobic glycolysis occurs in smooth muscle cells, endothelial cells, and fibroblasts in which oxidative phosphorylation is inhibited (Li et al., 2016). In summary, SCFA-producing bacteria were enriched in the MSC-treated group, and the function of the gut microbiota in the MSC-treated group was mainly focused on metabolic pathways. These results indicate that the gut microbiota of MSC-treated mice may treat PH by regulating metabolic pathways.

## Conclusion

In summary, our data indicate that MSCs can effectively treat hypoxia-induced PH in mice. The gut microbiota was changed in hypoxia-induced mice and tended to be similar to that under normoxia in MSC-treated mice. Our data also indicate that MSCs may treat hypoxia-induced PH mice by regulating metabolic pathways.

## Data Availability Statement

The datasets presented in this study can be found in online repositories. The names of the repository/repositories and accession number(s) can be found below: https://www.ncbi.nlm.nih.gov/, SRR14292713–SRR14292733.

## Ethics Statement

The studies involving human participants were reviewed and approved by the Institutional Review Board of the Shenzhen University General Hospital and the Institutional Review Board of the Shenzhen University School of Medicine. The patients/participants provided their written informed consent to participate in this study. The animal study was reviewed and approved by the Animal Care and Use Committee of the Shenzhen University. Written informed consent was obtained from the individual(s) for the publication of any potentially identifiable images or data included in this article.

## Author Contributions

DG conceived the study. LL designed the experiments, interpreted the results, and wrote the main manuscript text. LL and QC performed the experiments. LL and DG reviewed and edited the manuscript. All authors contributed to the article and approved the submitted version.

## Conflict of Interest

The authors declare that the research was conducted in the absence of any commercial or financial relationships that could be construed as a potential conflict of interest.

## Publisher’s Note

All claims expressed in this article are solely those of the authors and do not necessarily represent those of their affiliated organizations, or those of the publisher, the editors and the reviewers. Any product that may be evaluated in this article, or claim that may be made by its manufacturer, is not guaranteed or endorsed by the publisher.
