# Supplementary material for: MSCs Therapy Reverse the Gut Microbiota in Hypoxia-Induced Pulmonary Hypertension Mice
Source: Front Physiol. 2021 Aug 31;12:712139. doi: 10.3389/fphys.2021.712139 (PMC8438532; doi:10.3389/fphys.2021.712139)
Supplement: Supplementary file 1 [file Data_Sheet_1.docx]

Supplementary Material

**Supplementary Figure 1**

**
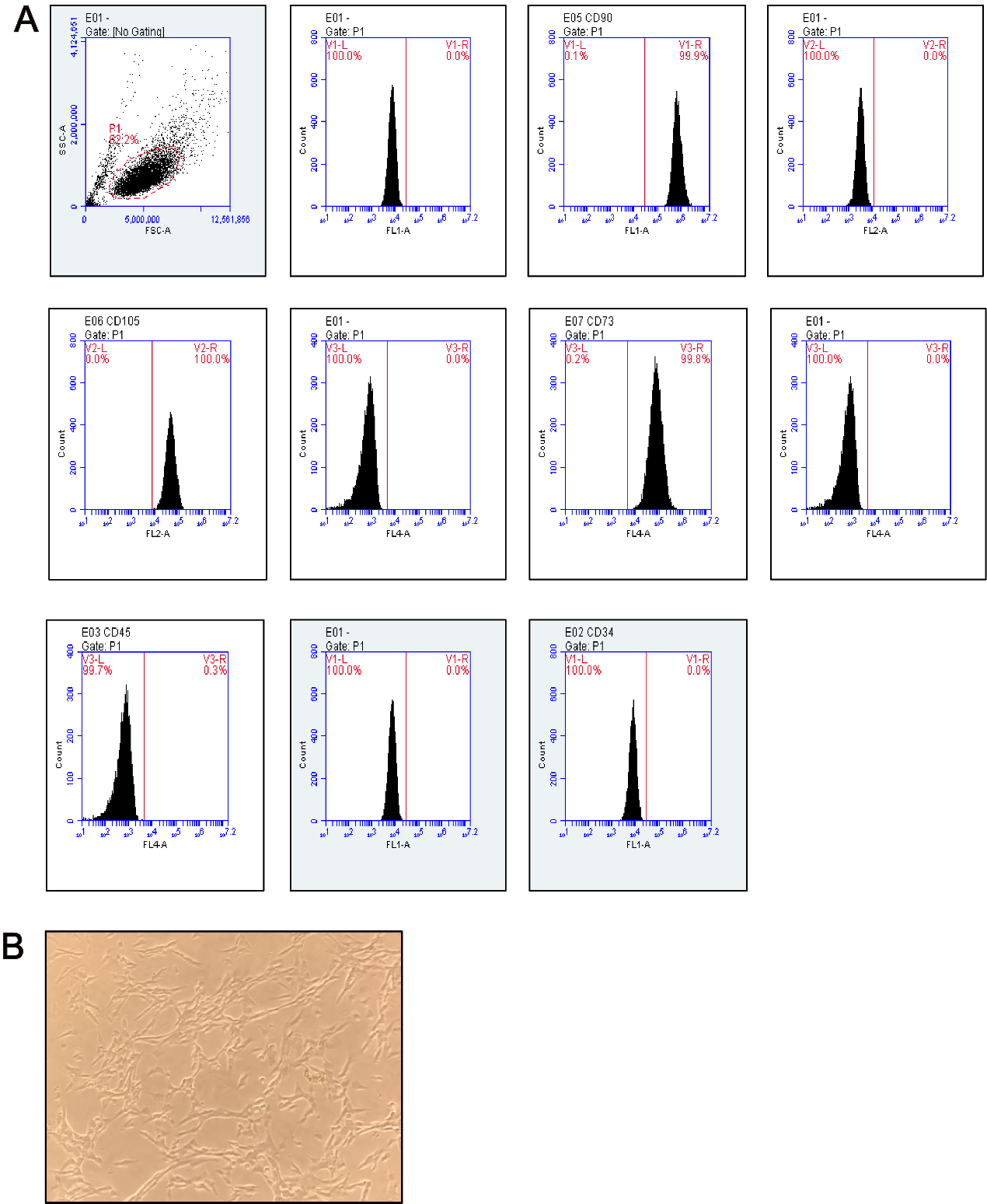
**

**Supplementary Figure 1** | **Immunophenotypic and morphology of Human umbilical cord blood-derived mesenchymal stem cells. (A),** Fluorescence-activated cell sorting results showed that MSCs were positive for CD90 (99.90 %), CD105 (100 %), and CD73 (99.8 %) and negative for CD45 (0.3 %), CD34 (0 %). **(B),** The classic spindle-shaped morphology of MSCs.

**Supplementary Figure 2**

**
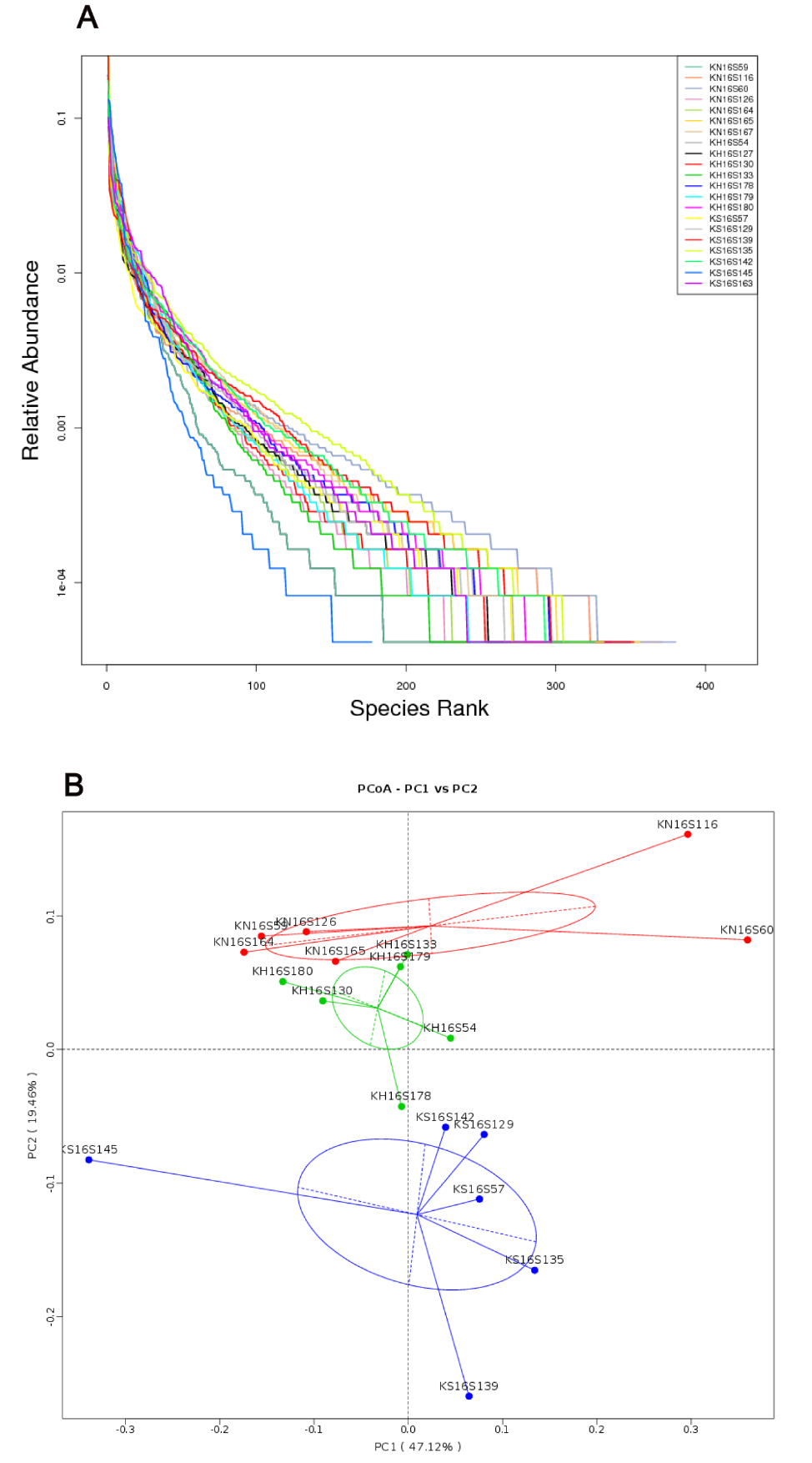
**

**Supplementary Figure 2 | The intestinal microbiota structure of normaxia, hypoxia-induced and MSC-treated mice. (A)**, Rank abundance curves. **(B)**, Principal coordinates analysis plot based on the UniFrac distance.

**Supplementary Table 1 |** Number of reads and operational taxonomic units, good coverage estimation, and diversity index for each sample from the pyrosequencing analysis.

|  | **#Sample_name** | **Clean_Reads(#)** | **Effective %** | **observed_species** | **shannon** | **simpson** | **chao1** | **ACE** | **goods_coverage** | **PD_whole_tree** |
| --- | --- | --- | --- | --- | --- | --- | --- | --- | --- | --- |
| **NC** | **KN16S59** | **80036** | **90.96** | **243** | **4.801** | **0.926** | **294.848** | **306.091** | **0.998** | **18.654** |
|  | **KN16S116** | **80211** | **94.43** | **371** | **5.655** | **0.948** | **403.667** | **399.372** | **0.998** | **25.77** |
|  | **KN16S60** | **80149** | **93.38** | **380** | **5.766** | **0.922** | **424.452** | **415.594** | **0.998** | **25.352** |
|  | **KN16S126** | **80089** | **93.82** | **285** | **5.667** | **0.962** | **353.077** | **337.373** | **0.998** | **21.435** |
|  | **KN16S164** | **80105** | **97.52** | **274** | **5.707** | **0.965** | **306.621** | **308.096** | **0.998** | **19.891** |
|  | **KN16S165** | **80110** | **96.7** | **356** | **6.042** | **0.968** | **413.037** | **392.955** | **0.998** | **25.284** |
|  | **KN16S167** | **80115** | **91.46** | **316** | **5.982** | **0.965** | **350.5** | **350.647** | **0.998** | **23.135** |
| **Hx** | **KH16S54** | **80066** | **94.7** | **332** | **5.354** | **0.929** | **397.357** | **377.232** | **0.997** | **24.347** |
|  | **KH16S127** | **80046** | **93.52** | **294** | **5.054** | **0.884** | **325.2** | **316.889** | **0.998** | **21.598** |
|  | **KH16S130** | **80078** | **96.55** | **309** | **5.609** | **0.961** | **349.923** | **348.64** | **0.998** | **22.616** |
|  | **KH16S133** | **80177** | **91.83** | **283** | **5.507** | **0.952** | **352.03** | **353.425** | **0.997** | **22** |
|  | **KH16S178** | **80077** | **92.09** | **350** | **5.671** | **0.946** | **379.118** | **391.018** | **0.998** | **25.524** |
|  | **KH16S179** | **80128** | **95.27** | **303** | **5.617** | **0.958** | **351.487** | **357.816** | **0.997** | **22.133** |
|  | **KH16S180** | **80128** | **96.23** | **337** | **6.024** | **0.968** | **390.323** | **381.067** | **0.998** | **23.749** |
| **Hx + MSC** | **KS16S57** | **80127** | **92.42** | **322** | **4.801** | **0.866** | **357.838** | **358.581** | **0.998** | **23.11** |
|  | **KS16S129** | **80236** | **94.18** | **321** | **5.76** | **0.96** | **372.333** | **362.289** | **0.998** | **22.227** |
|  | **KS16S139** | **80037** | **96.23** | **352** | **5.561** | **0.901** | **400.125** | **393.014** | **0.998** | **24.27** |
|  | **KS16S135** | **55507** | **97.99** | **332** | **6.545** | **0.98** | **343.118** | **347.707** | **0.999** | **23.159** |
|  | **KS16S142** | **80134** | **96.52** | **326** | **5.928** | **0.952** | **343.531** | **344.66** | **0.999** | **23.138** |
|  | **KS16S145** | **58175** | **97.22** | **177** | **4.706** | **0.935** | **187.969** | **198.293** | **0.999** | **14.453** |
|  | **KS16S163** | **54753** | **97.86** | **297** | **6.007** | **0.969** | **474.333** | **343.85** | **0.998** | **21.425** |

**Supplementary Table 2 |** Real time PCR primers used in this study.

| Primer | Sequence（5‘ - 3’） |
| --- | --- |
| GAPDH-F | **CTCATGACCACAGTCCATGC** |
| GAPDH-R | **TTCAGCTCTGGGATGACCTT** |
| iNos-F | **CAGCTCAAGAGCCAGAAACG** |
| iNos-R | **TTACTCAGTGCCAGAAGCTG** |
| IL1b-F | **CTTTGAAGAAGAGCCCATCC** |
| IL1b-R | **TTTGTCGTTGCTTGGTTCTC** |
| IL6--F | **CCAATTTCCAATGCTCTCCT** |
| IL6--F | **ACCACAGTGAGGAATGTCCA** |
| Cxcl10-F | **GCTGCAACTGCATCCATATCGATG** |
| Cxcl10-R | **TCCGGATTCAGACATCTCTGCTC** |
